# Effectiveness of Group Voice Therapy in Teachers with Hyperfunctional Voice Disorder

**DOI:** 10.3390/clinpract16010016

**Published:** 2026-01-14

**Authors:** Nataša Prebil, Rozalija Kušar, Maja Šereg Bahar, Irena Hočevar Boltežar

**Affiliations:** 1 Department of Otorhinolaryngology and Cervicofacial Surgery, University Medical Centre Ljubljana, 1000 Ljubljana, Sloveniairena.hocevar-boltezar@mf.uni-lj.si (I.H.B.); 2 Faculty of Education, University of Ljubljana, 1000 Ljubljana, Slovenia; 3 Faculty of Medicine, University of Ljubljana, 1000 Ljubljana, Slovenia

**Keywords:** voice disorders, teacher, voice therapy, group therapy, treatment outcomes

## Abstract

**Background/Objectives**: The aim of this study was to assess the short-term and long-term effectiveness of group voice therapy in changing vocal behaviour and improving voice quality (VQ) among teachers with hyperfunctional voice disorders (HFVD), using both subjective and objective measures. **Methods**: Thirty-one teachers participated in a structured group voice therapy programme. Participants underwent videoendostroboscopic evaluation of laryngeal morphology and function, perceptual assessment of voice, acoustic analysis of voice samples, and aerodynamic measurements of phonation. Patients’ self-assessment of VQ and its impact on quality of life were measured using a Visual Analogue Scale (VAS) and the Voice Handicap Index-30 (VHI-30). Evaluations were conducted at four time points: pre-therapy (T0), immediately post-therapy (T1), and at 3-month (T3) and 12-month (T12) follow-up visits. **Results**: Significant improvement was observed between T0 and T1 in perceptual voice evaluations: grade, roughness, asthenia, strain, loudness, fast speaking rate, as well as in neck muscle tension, shimmer, patients’ most harmful vocal behaviours, VHI-30 scores, patients VQ evaluation, and its impact on quality of life (all *p* < 0.05). Almost all parameters of subjective and objective voice assessment improved over the 12-month observation period, with the greatest improvement between T0 and T12 (all *p* < 0.05), indicating lasting reduced laryngeal tension and improved phonatory efficiency. **Conclusions**: Group voice therapy has been shown to be an effective treatment for teachers with HFVD, leading to significant and long-lasting improvements in perceptual, acoustic, and self-assessment outcomes. Therapy also promoted healthier vocal and lifestyle behaviours, supporting its role as a successful and cost-effective rehabilitation and prevention method for occupational voice disorders.

## 1. Introduction

Voice disorders disrupt communication, attract unwanted attention, and negatively affect both speaker and listener. The voice may be inappropriate for the speaker’s age, gender, or geographic background [1,2]. Such disorders result from improper vocal function (functional voice disorders) or organic laryngeal changes affecting normal function. Misuse or overuse causes excessive, uncoordinated muscle tension during phonation, leading to hyperfunctional voice disorder (HFVD). This can also contribute to the development of vocal nodules, polyps, or oedema on the vocal folds due to excessive collision at the contact point between them during vibration, which further increases maladaptive behaviour of the vocal folds [3].

Teachers are at higher risk of developing dysphonia and chronic voice disorders than the general population [4,5,6,7,8]. The main risk factors for voice disorders are unfavourable classroom conditions (noise, poor acoustics, humidity, or temperature), individual health conditions, and habits [9]. The occurrence of voice disorders in such professional voice users is of particular concern, as these disorders can significantly hinder the ability to perform teaching duties [10,11] and negatively affect overall quality of life [12,13].

The primary approach for treating HFVD is voice therapy [3], which is effective for the majority of patients [14]. There are no adopted common recommendations regarding the optimal programme, duration, or frequency of therapy sessions [15]. Voice therapy methods are classified as indirect or direct [16,17,18,19]. Indirect intervention includes counselling about lifestyle, vocal habits, and other vocal hygiene instructions [18]. Direct voice interventions aim to replace disordered vocal technique with vocal behaviours that provide a sustainable, functional, and efficient voice [20]. Voice therapy can be performed on a one-to-one basis (patient–therapist) or as group therapy (group of individuals with similar disorders–therapist) [21].

Reports on the effectiveness of group therapy have emerged mainly over the past two decades, showing promising results for patients with various voice disorders. Several studies have demonstrated its effectiveness in improving voice quality, reducing vocal symptoms and personal risk factors, and decreasing anxiety levels [22,23,24,25]. Additionally, research has shown significant benefits for professional voice users, particularly in improving work productivity and voice quality [26]. Several studies report that group voice therapy is as effective as individual therapy in professional voice users with HFVD [21], as well as in individuals from the general population with functional [27] and various other voice disorders [28]. The effectiveness of voice therapy is typically measured by comparing pre- and post-treatment findings from videolaryngostroboscopy, expert auditory-perceptual evaluation, acoustic and aerodynamic measurements, and patient self-assessment of voice [29,30]. European guidelines for the assessment of voice quality (VQ) include videolaryngostroboscopy, patient-reported VQ (Voice Handicap Index with 30 items—VHI-30 or with 10 items—VHI-10), perceptual assessment of voice (Grade, Roughness, Breathiness, Asthenia and Strain—GRBAS scale), aerodynamic measurements (maximum phonation time—MPT), and acoustic analysis of voice samples (mean F0, jitter, shimmer, and noise-to-harmonic ratio—NHR) [31]. The primary goal of voice therapy is to achieve long-term changes in vocal behaviour and phonation technique in everyday life. To determine whether this goal has been met, follow-up testing after therapy is essential—typically around one year later, when the patient has been exposed to daily vocal demands at work without direct therapist supervision [27]. There are some studies on the assessment of the effectiveness of individual voice therapy, but very few on the effectiveness of group therapy. Only a few studies have shown the success of group therapy immediately after therapy [21], and one year post-therapy [28]. The study by Ohlsson et al. (2018) also included an intermediate evaluation at three months after therapy [27]. Published studies on the effectiveness of individual and/or group voice therapy have most commonly used the validated VHI questionnaire [21,27,28], patient self-assessment of VQ on a visual analogue scale (VAS) [27], expert perceptual ratings using the GRBAS scale [21,27], and objective acoustic and aerodynamic phonation measurements [21,27] for evaluation. To the best of our knowledge, only one study has combined subjective and objective methods to evaluate group voice therapy effectiveness in terms of both improved VQ and reduced patient handicap [27]. The study included women and men who were consecutive patients on a waiting list for voice therapy for functional voice disorder, without specifying their profession. The quality of their voices was assessed subjectively by the participants themselves and by a speech-language pathologist using a visual analogue scale. The VHI questionnaire was used to measure the handicap caused by the voice disorder. Objective voice assessment was conducted using a voice range profile [27].

The aim of the present study was to focus on a vulnerable group of professional voice users—teachers with HFVD—to assess the effectiveness of group voice therapy in changing vocal behaviour and improving VQ among teachers with HFVD using both subjective patients and expert assessments, as well as objective measurements. In comparison with Ohlsson’s study [27], we included only teachers, expanded the objective assessment to include some aerodynamic measurements, and for the expert subjective assessment of the voice, the GRBAS scale was used. Effectiveness was evaluated not only immediately after therapy, but also at three months and one year post-therapy to assess both short-term and long-term outcomes.

## 2. Materials and Methods

### 2.1. Participants

Consecutive teachers who attended our out-patient phoniatric department for voice problems between 2023 and 2024 were invited to participate in the study. Inclusion criteria were HFVD diagnosed by phoniatric examination of the larynx, employment as a professional voice user (teacher in preschool, primary, or secondary school), being on a waiting list for voice therapy, and signed informed consent for participation. Patients both without and with mucosal lesions on the vocal folds resulting from HFVD were included. Exclusion criteria were previous surgical treatment for any laryngeal disease and any prior voice therapy for voice disorders. After considering all inclusion and exclusion criteria, the study included 34 teachers diagnosed with HFVD, all of whom were female. Three patients did not complete the entire protocol. At the end, there were 31 women in the study. Participants ranged in age from 26 to 58 years, with a mean age of 39.5 years and a standard deviation (SD) of 6.7 years.

### 2.2. Study Protocol

All assessments were performed before inclusion in the programme (T0), at the end of the programme (T1), and at both follow-up sessions at 3 months (T3) and 12 months (T12) after completion of therapy. The participants underwent a routine otorhinolaryngological phoniatric examination with videoendostroboscopy to assess morphological vocal fold lesions (no lesions, vocal nodules, polyp, oedema, or other) and laryngeal function during phonation (presence or absence of signs of HFVD based on disordered vocal fold vibration, incomplete vocal folds closure, and disordered mucosal wave).

An independent, experienced clinical speech-language pathologist, who did not participate in the group therapy, performed clinical perceptual voice assessments. Loudness was rated perceptually during sustained phonation and spontaneous speech and categorised as excessive, adequate, or too soft based on clinical judgement. Speaking rate was assessed during spontaneous speech and classified as fast or adequate according to typical conversational speech. Neck muscle tension during speaking was assessed by visual inspection and palpation of the extrinsic laryngeal and neck muscles and categorised as excessive or adequate. VQ was evaluated using the GRBAS scale (Grade, Roughness, Breathiness, Asthenia, Strain) on a 4-point scale (0 = normal, 1 = mild, 2 = moderate, 3 = severe pathology) [29].

Acoustic analysis of sustained vowel/a/was performed with the Multi-Dimensional Voice Program (KayPentax, Lincoln Park, NJ, USA), measuring fundamental frequency (F0), jitter, and shimmer. Aerodynamic measures included MPT and the ratio of maximal production of unvoiced to voiced consonant (*s*/*z* ratio). All measurements were performed three times, and the mean values of the three measurements were used for statistical analysis. In the case of MPT, the longest value was used for further analysis. Participants completed the VHI-30 questionnaire [32], validated in Slovenian [33], which contains functional, physical, and emotional domains. Additionally, participants independently completed a questionnaire addressing voice risk factors (smoking, harmful habits affecting voice quality such as loud speaking, shouting, fast speaking rate, excessive talking, and throat clearing, insufficient hydration, and vocal load outside work). Current voice-related symptoms (vocal fatigue, dry throat, throat pain, globus sensation during talking, and voice loss) were also recorded. They marked each factor as present (often/always) or absent (never/rarely). They rated their VQ and its impact on quality of life on a Visual Analogue Scale (VAS; range 0–10).

### 2.3. Group Therapy Program

The intervention comprised nine group therapy sessions: seven weekly therapy sessions of 90 min each, and two follow-up assessment sessions. The rehabilitation protocol followed a general scheme routinely used in our clinical service and integrated both indirect and direct voice therapy approaches, as commonly applied in the management of HFVD. The programme incorporated voice therapy techniques traditionally used in our region and routinely applied by the clinicians involved, who were formally trained and had extensive clinical experience in rehabilitation of voice disorders. This approach ensured clinical feasibility, consistency of intervention delivery, and ecological validity of the group therapy programme. Groups consisted of 4–5 participants.

- Sessions 1–2: Introduction and education on normal voice production and common voice pathologies; collection of participants’ voice histories; structured education on vocal hygiene, including voice load management, hydration, avoidance of vocally harmful behaviours, and lifestyle-related risk factors for dysphonia. Based on individual vocal demands and self-reported habits, personalised vocal hygiene recommendations were provided.
- Sessions 3–4: Review of hygiene adherence, training and practice of abdominal breathing techniques, muscle warm-ups (tongue, lips, jaw, shoulders, neck, head posture), vocal fold warm-ups (lip/trill and tongue trill), and vocal tract relaxation exercises (yawning, massage).
- Sessions 5–7: Review of hygiene adherence, continuation of previous exercises with the addition of semi-occluded vocal tract exercises. Carry-over into functional speech was systematically supported within the group setting through guided verbal interaction, spontaneous speech tasks, and group discussion, with continuous therapist feedback aimed at promoting efficient phonation and reducing laryngeal tension in everyday voice use.
- Sessions 8 and 9: Follow-up voice assessments performed three months and twelve months after therapy completion.

### 2.4. Statistical Analysis

Statistical analyses were conducted using IBM SPSS Statistics 28. The results of perceptual voice assessment, acoustic analysis of voice samples, aerodynamic measurements, questionnaire results on risk factors, VHI-30, patient self-assessment of voice, and the impact of VQ on quality of life were compared between each pair of consecutive time points (T0–T1, T1–T3, T3–T12), and between T0 and T12. Comparisons between measurements at different time points were performed using the χ^2^ test (Fisher’s exact test where appropriate), paired-samples *t*-test, and paired Mann-Whitney U tests. Statistical significance was determined at *p* < 0.05. In cases of significance, the effect size was presented as Cohen’s *d* or Cramer’s *V*.

### 2.5. Ethical Aspects

The study was conducted in accordance with the World Medical Association Code of Ethics (Declaration of Helsinki) and was approved by the National Medical Ethics Committee of the Republic of Slovenia (Document No. 0120-412/2022/4, dated 8 December 2022). Each participant signed informed consent before participating in the study.

## 3. Results

### 3.1. Participants’ Characteristics and Voice Problems

The majority of the participants were primary school teachers with less than 10 years of experience in the educational process. Data on their length of career and employment setting are presented in Table 1.

Voice problems during their careers were reported by 16 participants (51.6%), with 3 (9.7%) experiencing them frequently, and 12 (38.7%) reporting no significant voice problems until the present voice disorder. The duration of voice problems before attending the phoniatric department ranged from 0 to 156 months (mean 24.2 months, SD 34.0 months). Most participants experienced multiple concurrent symptoms before undergoing voice therapy, and vocal overload was the main reason for their voice problems (Table 1).

Eighteen participants (58.1%) reported engaging in vocally demanding activities during their free time. All participants were non-smokers at the time of the study; however, one teacher had quit smoking more than five years before participating in the study. Three participants (9.7%) had attended speech therapy for sigmatism in childhood.

### 3.2. Videoendostroboscopic Findings

Before group therapy, HFVD was detected in all teachers based on irregular vocal fold vibration, disordered mucosal wave (15 patients, 48.4%), and/or mucosal lesions, which are typical consequences of HFVD (22 patients, 71.0%). Vocal fold nodules were found in 18 patients (58.1%), a flat polyp in 2 patients (6.5%), and slight vocal folds oedema in 2 patients (6.5%). Nine patients (29%) had HFVD without mucosal lesions on the vocal folds. Immediately after therapy, vocal fold lesions were seen in 4 patients (T0–T1: *p* = 0.000), at T3 in 3 patients (T1–T3: *p* = 1.000), and at T12 in 2 patients (T3–T12: *p* = 0.667; T0–T12: *p* = 0.000). After therapy, disordered vocal fold vibration was observed in 6 patients at T1 (T0–T1: *p* = 0.016), in 5 patients at T3 (T1–T3: *p* = 0.785), and in 4 patients at T12 (T3–T12: *p* = 0.728; T0–T12: *p* = 0.005).

### 3.3. Perceptual Voice Evaluation

An experienced speech-language pathologist conducted a perceptual evaluation of patients’ VQ using the GRBAS scale, loudness, and speaking rate, as well as a clinical assessment of cervical muscle tension at four time points. Almost all voice characteristics showed significant improvement immediately after completion of group voice therapy, and all parameters demonstrated significant improvement at T12 compared with T0. The calculations of effect size showed varying practical significance, ranging from small (*d* > 0.2) to moderate (*d* > 0.5) or strong (*d* ≥ 0.8) in GRBAS assessments, but very strong (*V* > 0.25) in the perceptual evaluation of loudness, speaking rate, and cervical muscle tension (Table 2).

### 3.4. Acoustic and Aerodynamic Analysis

Significant improvements were observed in jitter (between T1 and T3, and between T0 and T12) and in shimmer (between T0 and T1). Before treatment and immediately after therapy, the mean jitter value was above the threshold specified by the instrument manufacturer. Three months after therapy, the mean value decreased below the threshold and remained within normal limits even one year after completing group therapy. F0 almost did not change at four time points. Shimmer was within the normal range before and after therapy. The *s*/*z* ratio also indicated significantly more efficient respiratory-phonatory coordination as an improvement between T0 and T12. The calculations of effect size showed that practical significance was small (*d* > 0.2) to moderate (*d* > 0.5) in all cases. Detailed results are presented in Table 3.

### 3.5. Voice-Related Behaviors and Lifestyle Factors

Using the same questionnaire, the participants evaluated the presence of their own voice-related behaviours that could affect voice quality at four time points. Significant improvements were observed in shouting, fast speech, and throat clearing immediately after therapy and at T12. Loud speaking did not change significantly at any time point, and excessive talking improved only after twelve months compared to baseline. For unfavourable lifestyle factors, significant reductions were found in sleep deprivation and insufficient hydration immediately after therapy and at T12. The calculations of effect size showed that practical significance was very strong (*V* > 0.25) in all cases. Detailed results are presented in Table 4.

### 3.6. Self-Assessment of Voice Quality and Quality of Life (VHI and VAS)

Significant improvement was observed in VHI scores across all domains following therapy. The mean total VHI score decreased markedly up to three months after therapy, with values remaining stable at T12. Among the subscales, the physical (VHI-P) and emotional (VHI-E) domains showed consistent improvement during the initial three months and maintained these reduced scores thereafter, while the functional (VHI-F) subscale demonstrated statistically significant changes between T1 and T3, and between T0 and T12. The calculations of effect size showed varying practical significance, ranging from small (*d* > 0.2) to moderate (*d* > 0.5) or strong (*d* > 0.8). No significant differences were found between the T3 and T12 assessments in any of the VHI domains (Table 5).

Regarding the assessment of self-perceived voice quality and its impact on quality of life using the VAS, participants reported decreased handicap associated with voice disorders and increased overall satisfaction with their voice and quality of life over time. See Table 5.

## 4. Discussion

The present study evaluated the effectiveness of group voice therapy in professional voice users—teachers with HFVD. To the best of our knowledge, this is the first study to provide such comprehensive subjective and objective evaluations of the effectiveness of group voice therapy in patients with functional voice disorder, including a one-year follow-up after treatment. The methods used in the study depended on the availability of equipment in our institution and the use of comparable methods in other studies addressing the success of voice therapy. The results demonstrated significant improvement in perceptual, acoustic, aerodynamic, and self-assessed voice parameters, confirming that structured group-based therapy is an effective and sustainable intervention for this population.

In Slovenia, the prevalence of voice disorders among teachers is a significant problem. The only study on the prevalence of voice problems in teachers included 1509 subjects (13% males). The prevalence of voice problems in the current year was 66% (occasionally 51%, frequently 15%), and over the entire career 89% (occasionally 71%, frequently 18%). The reasons for such a high prevalence are probably the lack of courses on vocal health care during the educational process for future teachers, the large number of pupils in each class, the high number of teaching hours per week and per day, and a shortage of teachers. An otorhinolaryngological examination, or at least a screening test of vocal abilities before entering the educational programme, is not required for prospective teachers [34].

At the beginning of the study, mucosal lesions on the vocal folds were observed in 22 (71%) teachers, and obvious disordered vocal fold vibration in 15 (48.4%) patients. Improvement in the presence of mucosal lesions (nodules, polyps, or oedema) on vocal folds resulting from excessive forces at the collision of the vocal folds during vibration, was demonstrated immediately after treatment, as these lesions disappeared in 20 out of 22 teachers and laryngeal function normalised in 9 out of 15 teachers. One year after completing therapy, little vocal fold nodules were seen in only 2 (6.5%) participants, and disordered vocal fold vibration in an additional 2 (6.5%) teachers, demonstrating that 87% of teachers retained proper phonation technique even without speech-language pathologist supervision.

The most notable perceptual improvements were observed in the expert evaluation parameters G, R and S, while self-assessment results (VHI-30 and VQ assessment on VAS) indicated a progressive reduction in perceived voice handicap and increased satisfaction with VQ. Acoustic and aerodynamic measurements showed corresponding trends, with significant decreases in jitter and shimmer and an improved *s*/*z* ratio, reflecting greater vibratory stability and more efficient coordination between respiration and phonation. Importantly, most improvements were maintained up to twelve months after the completion of therapy, supporting the long-term benefits of group intervention.

Significant reductions in VHI scores after group voice therapy were confirmed in several earlier studies [21,23,27,28]. In particular, the study by Ohlsson et al. [27] demonstrated significant improvements in the VHI total and subscale scores, as well as in self-ratings of hoarseness and vocal fatigue, both immediately after therapy and at 3- and 12-month follow-up evaluations. This finding is consistent with the present results and emphasises that group voice therapy can produce durable and clinically meaningful improvements in self-perceived vocal function. The inclusion of a 12-month follow-up in both studies provides important evidence of long-term therapy effectiveness, addressing one of the key limitations of earlier research, which typically assessed outcomes only immediately or shortly after therapy.

However, unlike in Ohlsson’s study [27], where the perceptual evaluation of voice by speech-language pathologists did not show significant improvement after group therapy, our results revealed marked perceptual gains across all GRBAS parameters following group intervention. The most notable and statistically significant improvements were found in G, R, and S, indicating that group therapy in our programme effectively reduced laryngeal tension during phonation and improved overall VQ (Table 2). The same three parameters, G, R, and S, also showed significant post-therapy improvement in the study by Alam et al. [21], further supporting the effectiveness of group-based intervention for individuals with HFVD. Our findings are also largely consistent with those of Cantarella et al. [23], who reported significant post-therapy improvements in perceptual assessment of VQ. In both studies, the most notable perceptual improvements were observed in the parameters G and R, reflecting enhanced overall VQ and reduced irregularity of vocal fold vibration. While Cantarella and colleagues [23] found the third strongest improvement in B, indicating better glottal closure and reduced air leakage, the current study revealed the most pronounced changes in S, reflecting a substantial reduction in excessive laryngeal tension during voice production. This difference may be explained by the specific characteristics of the participant group, which consisted exclusively of educational professionals with HFVD, in whom excessive muscular effort and constriction were predominant pathophysiological features. The more pronounced reduction in S in our study may also be attributed to concurrent physiological and morphological changes observed during clinical examination. At the initial assessment, neck muscle tension during speech was noted in 16 participants (51.6%), whereas after twelve months it was observed in only two subjects (6.5%). This finding suggests that the therapy effectively reduced both laryngeal and extra-laryngeal hyperfunction, confirming that participants achieved a more relaxed and efficient phonatory pattern over time. The alignment between clinical observations and perceptual ratings further strengthens the evidence for the overall therapeutic success.

Acoustic and aerodynamic measures showed corresponding trends, with decreases in jitter and shimmer and an improved *s*/*z* ratio, indicating greater vibratory stability and more efficient coordination between respiration and phonation. Jitter significantly decreased between the T1 and T3 assessments and returned to normal. It remained within the normal range even at T12. Shimmer values were within normal limits at all measurement points; however, they showed significant improvement immediately after therapy. The *s*/*z* ratio improved significantly between T0 and T12, with a gradual positive trend across all time points. These findings are partly consistent with those of Alam et al. [21], who reported significant post-therapy reductions in jitter and shimmer after group intervention. Unlike in Alam’s study, which assessed outcomes only immediately after therapy, our results demonstrated a delayed but sustained improvement in jitter and long-term stabilization of acoustic parameters. The persistence of these changes up to twelve months supports the lasting benefits of group voice therapy in enhancing vocal stability and respiratory–phonatory coordination.

F0 was at the lower limit of the normal range for female voices and did not change significantly during the study. As all included teachers were non-smokers, the slightly lower F0 could be due to anatomical laryngeal characteristics of the subjects or very mild oedema of the vocal folds resulting from the participants’ heavy vocal load. If vocal overload were the reason for the lower F0, an increase in F0 would be expected after improved phonation technique following treatment, but this was not observed.

In evaluating the meaning of acoustic parameters, we must bear in mind that they are measured on short vowel samples and can serve only as a method to monitor the success of treatment. Perceptual assessment of voice by a speech-language pathologist during conversation provides a much more comprehensive evaluation of voice quality than acoustic analysis of just a few seconds of vowel phonation.

In addition to perceptual and acoustic improvements, participants showed meaningful changes in lifestyle factors. Immediately after therapy, and again at the twelve-month follow-up, there were significant reductions in unfavourable voice use habits (shouting, fast speech, and throat clearing), indicating improved knowledge of vocal hygiene and greater awareness of harmful vocal behaviours. Excessive talking decreased significantly only after twelve months, suggesting that some behavioural adjustments may require longer consolidation periods once participants return to their regular teaching routines. Loud speaking did not change significantly at any time point, which likely reflects the professional reality of teaching, large classrooms, background noise, poor room acoustics, and the widespread belief among educators that effective teaching requires increased vocal intensity. At baseline, 38.79% of participants used excessive loudness in communication with the speech-language therapist. After therapy, only 3 participants (9.7%) exhibited this behaviour, and at T12, only 2 subjects were assessed as speaking too loudly in one-to-one communication. We presume that the classroom environment and the large number of pupils in the class compel teachers to use excessive loudness during their work. As education on voice care is not part of the curriculum for future teachers in Slovenia, it is possible that a lack of important information about the harmful effects of excessive loudness on the vocal folds is another cause of improper vocal behaviour in the classroom. We believe that including at least a short course on voice hygiene in the curriculum for future teachers would improve their vocal habits.

Significant improvements were also observed in sleep hygiene and hydration immediately after therapy and again at twelve months compared with baseline, consistent with increased adherence to vocal hygiene education. Da Silva et al. [24] also reported a significant post-therapy reduction in personal vocal risk factors, including vocally abusive behaviours (shouting, throat clearing) and insufficient hydration. These results emphasise that group voice therapy not only improves phonatory function but also fosters positive behavioural and lifestyle changes that contribute to the prevention of future voice problems. It is possible that therapy in a group of subjects with the same profession and similar voice problems encourages group dynamics, with mutual support on one hand and competition in adhering to hygiene norms on the other. It may also result from the idea of a group task, making participants more willing to continue proper voice exercises long after completing therapy.

The main limitation of this study is the relatively small sample size and the absence of a control group receiving individual therapy. Future studies should therefore include direct comparisons between group and individual therapy to further clarify relative efficacy and to identify factors that contribute to the long-term maintenance of therapeutic outcomes in professional voice users. Given that a large number of professional voice users with voice disorders require therapy to continue working, group therapy—which is at least comparably effective to individual therapy and less costly, as it includes more patients simultaneously—could help meet the high demand for voice treatment.

Another limitation is that the study participants were exclusively women. As there are few men working as teachers in nursery, primary, or secondary schools in Slovenia, and most candidates for voice therapy are women, these factors may explain why only women were included. In addition, female gender was identified as a risk factor for voice problems in teachers in Slovenia [34]. Therefore, we can limit our conclusions about the effectiveness of group voice therapy to female teachers only.

We are aware that measuring MPT and the *s*/*z* ratio is a limitation in the precise assessment of the aerodynamic capabilities of the patients, and is subject to certain influencing factors. The *s*/*z* ratio, as a task of maximum performance of laryngeal valving during phonation, is particularly limited by inter- and intra-individual variability related to fatigue, learning effects, and the inherent difficulty of task replication [35]. There are also better, more modern methods for acoustic analysis of voice samples than were used in our study. In selecting the methods, we followed the guidelines of the European Laryngological Society and the Union of European Phoniatricians [31], and used methods from previous comparable studies, but were limited by the equipment available in our hospital.

Another limitation relates to the assessment of loudness, speaking rate, and neck muscle tension, which were based on structured clinical perceptual judgement rather than instrumental measurements. Although such assessments reflect routine clinical practice and were performed by an experienced speech-language pathologist, the absence of objective measures (e.g., sound pressure level in dB SPL or instrumental assessment of muscle activity) may introduce a degree of subjectivity and limit inter-rater comparability. Future studies should incorporate objective measures of vocal intensity and muscle tension to further strengthen methodological robustness.

## 5. Conclusions

Group voice therapy proved to be an effective intervention for female educational professional voice users with HFVD, leading to significant and sustained improvements in perceptual, acoustic, and self-assessed voice outcomes. The therapy, probably through group dynamics, also promoted healthier vocal behaviours and lifestyle habits, contributing to long-term vocal well-being. These findings support the use of structured group programmes as a cost-effective and sustainable approach to the prevention and rehabilitation of occupational voice disorders.

## Figures and Tables

**Table 1:** Baseline occupational and voice-related characteristics of participants before therapy in 31 teachers with hyperfunctional voice disorders.

Variable	Category Yes *n* (%)
**Length of career**	
Up to 10 years	17 (54.1)
11 to 20 years	7 (22.6)
21 to 30 years	4 (12.9)
**Employment setting**	
Preschool	12 (38.7)
Primary school	16 (51.6)
Secondary school	3 (9.7)
**Cause of current voice problems**	
Vocal overload	20 (64.5)
Upper respiratory tract infection	2 (6.5)
Upper respiratory tract infection and vocal overload	9 (29)
**Voice-related complaints before therapy**	
Vocal fatigue	26 (83.9)
Dry Throat	22 (71.0)
Pain when speaking	21 (67.7)
Sensation of a lump in the throat when speaking	21 (67.7)
Voice loss	13 (41.9)

**Table 2:** Comparison of speech-language pathologist’s assessment of voice quality (Grade, Roughness, Breathiness, Asthenia, and Strain), loudness, and speaking rate at four time points in 31 teachers with hyperfunctional voice disorder.

Parameter	T0 Mean ± SD or *N* (%)	T1 Mean ± SD or *N* (%)	T3 Mean ± SD or *N* (%)	T12 Mean ± SD or *N* (%)	*p*/*d* or *V* (T0–T1)	*p*/*d* or *V* (T1–T3)	*p*/*d* or *V* (T3–T12)	*p*/*d* or *V* (T0–T12)
Grade (G)	1.35 ± 0.75	0.77 ± 0.72	0.55 ± 0.51	0.52 ± 0.63	**0.000/0.79**	**0.035/0.35**	0.763	**0.000/1.19**
Roughness (R)	1.03 ± 0.75	0.52 ± 0.63	0.23 ± 0.43	0.39 ± 0.62	**0.002/0.74**	**0.013/0.54**	0.166	**0.000/0.93**
Breathiness (B)	0.55 ± 0.72	0.32 ± 0.60	0.10 ± 0.30	0.06 ± 0.25	0.090	0.070	0.564	**0.001/0.91**
Asthenia (A)	0.23 ± 0.43	0.06 ± 0.25	0.00 ± 0.00	0.03 ± 0.18	**0.025/0.48**	0.157	0.317	**0.014/0.61**
Strain (S)	0.81 ± 0.65	0.29 ± 0.46	0.03 ± 0.18	0.19 ± 0.40	**0.001/0.92**	**0.005/0.74**	0.025	**0.000/1.15**
Excessive	12 (38.7)	3 (9.7)	3 (9.7)	2 (6.5)	**0.016/0.34**	1.000	0.346	**0.000/0.44**
loudness								
Fast speaking rate	10 (32.3)	1 (3.2)	1 (3.2)	**0**	**0.006/0.38**	1.000	0.230	**0.000/0.44**
Excessive								
cervical muscle tension	16 (51.6)	2 (6.5)	1 (3.2)	2 (6.5)	**0.000/0.49**	1.000	1.000	**0.000/0.50**

Bold *p*-values indicate statistically significant differences (*p* < 0.05). Time points: T0 = before therapy, T1 = immediately after therapy, T3 = 3 months post-therapy, T12 = 12 months post-therapy. SD = standard deviation; *d* = Cohen’s *d*, *V* = Cramer’s *V*.

**Table 3:** Changes in acoustic and aerodynamic parameters at four time points in 31 teachers with hyperfunctional voice disorder.

Parameter	T0 (Mean ± SD)	T1 (Mean ± SD)	T3 (Mean ± SD)	T12 (Mean ± SD)	*p*/*d* (T0–T1)	*p*/*d* (T1–T3)	*p*/*d* (T3–T12)	*p*/*d* (T0–T12)
F0 (Hz)	199.99 ± 21.53	205.73 ± 18.84	204.04 ± 15.43	203.99 ± 19.58	0.135	0.629	0.988	0.619
Jitter (%)	1.41 ± 0.82	1.22 ± 0.86	0.88 ± 0.44	0.89 ± 0.47	0.071	**0.014/0.49**	0.702	**0.000/0.78**
Shimmer (%)	3.05 ± 1.28	2.55 ± 1.23	2.5 ± 0.85	2.64 ± 0.90	0.006	0.906	0.399	0.052
MPT (s)	15.39 ± 6.59	16.65 ± 7.20	16.75 ± 7.19	16.22 ± 6.45	0.090	0.905	0.409	0.363
*s*/*z* ratio	1.27 ± 0.27	1.24 ± 0.25	1.20 ± 0.25	1.17 ± 0.17	0.619	0.345	0.317	**0.033/0.44**

Bold *p*-values indicate statistically significant differences (*p* < 0.05). Time points: T0 = before therapy, T1 = immediately after therapy, T3 = 3 months post-therapy, T12 = 12 months post-therapy. SD = standard deviation. F0 = fundamental frequency; MPT = maximum phonation time; *d* = Cohen’s *d*.

**Table 4:** Comparison of the presence of personal vocal risk factors (voice-related behaviours and lifestyle factors according to the questionnaire) at four time points in 31 teachers with hyperfunctional voice disorders.

Behaviour	T0 *n* (%)	T1 *n* (%)	T3 *n* (%)	T12 *n* (%)	*p*/*V* (T0–T1)	*p*/*V* (T1–T3)	*p*/*V* (T3–T12)	*p*/*V* (T0–T12)
Loud speaking	29 (90.3)	26 (83.9)	24 (77.4)	25 (80.6)	0.707	0.523	0.755	0.279
Shouting	22 (71.0)	8(25.8)	9 (29.0)	5(16.1)	**0.000/0.45**	0.776	0.224	**0.000/0.55**
Fast speech	26 (83.9)	18 (58.1)	19 (61.3)	19 (61.3)	**0.025/0.28**	0.736	1.000	**0.046/0.25**
Excessive talking	31(100.0)	27 (87.1)	24 (77.4)	27 (87.1)	0.113	0.508	0.313	**0.033/0.26**
Throat clearing	16 (51.6)	3 (9.7)	5 (16.1)	7 (22.6)	**0.001/0.45**	0.707	0.502	**0.018/0.30**
**Lifestyle**								
Sleep deprivation	25 (80.6)	16 (51.6)	17 (54.8)	13 (41.9)	**0.016/0.31**	0.799	0.309	**0.002/0.39**
Insufficient hydration	23 (74.2)	8 (25.8)	15 (48.4)	14 (45.2)	**0.000/0.49**	0.066	0.733	**0.020/0.29**

Bold *p*-values indicate statistically significant differences (*p* < 0.05). Time points: T0 = before therapy, T1 = immediately after therapy, T3 = 3 months post-therapy, T12 = 12 months post-therapy. *V* = Cramer’s *V*.

**Table 5:** Changes in Voice Handicap Index (VHI) and Visual Analogue Scale (VAS) scores at four time points in 31 teachers with hyperfunctional voice disorder.

Parameter	T0 (Mean ± SD)	T1 (Mean ± SD)	T3 (Mean ± SD)	T12 (Mean ± SD)	*p*/*d* (T0–T1)	*p*/*d* (T1–T3)	*p*/*d* (T3–T12)	*p*/*d* (T0–T12)
VHI	45.84 ± 17.48	33.32 ± 15.74	26.58 ± 13.35	22.97 ± 16.42	**0.000/0.96**	**0.004/0.66**	0.133	**0.000/0.35**
VHI-F	9.87 ± 5.53	9.35 ± 4.88	7.1 ± 4.08	5.84 ± 5	0.508	**0.007/0.50**	0.135	**0.009/0.76**
VHI-P	22.06 ± 8.36	15.16 ± 6.65	12.84 ± 6.31	11.84 ± 7.89	**0.000/0.91**	**0.018/0.36**	0.545	**0.000/1.25**
VHI-E	13.9 ± 7.47	8.81 ± 5.88	6.65 ± 4.90	5.29 ± 5.13	**0.001/0.76**	**0.015/0.47**	0.082	**0.000/1.34**
VAS	5.16 ± 1.70	7.17 ± 1.13	7.48 ± 1.26	8 ± 1.34	**0.000/1.09**	0.178	0.107	**0.000/1.57**
VAS-QoL	4.69 ± 2.14	2.63 ± 1.68	2.25 ± 1.59	1.58 ± 1.87	**0.000/0.35**	0.314	0.056	**0.000/0.53**

Bold *p*-values indicate statistically significant differences (*p* < 0.05). Time points: T0 = before therapy, T1 = immediately after therapy, T3 = 3 months post-therapy, T12 = 12 months post-therapy. VHI—Voice Handicap Index; VHI-F—functional subscale; VHI-P—physical subscale; VHI-E—emotional subscale; VAS—Visual Analogue Scale; VAS-QoL—perceived impact of voice disorder on quality of life; SD = standard deviation; *d* = Cohen’s *d*.

## Data Availability

The datasets presented in this article are available from the corresponding author on personal requests.

## Abbreviations

The following abbreviations are used in this manuscript:

VQ	Voice quality
HFVD	Hyperfunctional voice disorders
VAS	Visual Analogue Scale
VHI-30	Voice Handicap Index-30
T0	Pre-therapy voice evaluation
T1	Immediately post-therapy follow-up visit
T3	3-month follow-up visit
T12	12-month follow-up visit
VHI-10	Voice Handicap Index-10
GRBAS	Grade, Roughness, Breathiness, Asthenia and Strain
MPT	Maximum phonation time
F0	Fundamental frequency
NHR	Noise-to-harmonic ratio
